# Diagnosis of monogenic diabetes: 10‐Year experience in a large multi‐ethnic diabetes center

**DOI:** 10.1111/jdi.12432

**Published:** 2015-10-26

**Authors:** Ellen RA Thomas, Anna Brackenridge, Julia Kidd, Dulmini Kariyawasam, Paul Carroll, Kevin Colclough, Sian Ellard

**Affiliations:** ^1^ Guy's and St Thomas’ NHS Foundation TrustLondonUK; ^2^ Royal Devon and Exeter HospitalExeterUK

**Keywords:** Diabetes mellitus, Genetic testing, Hepatocyte nuclear factor 1‐alpha

## Abstract

**Aims/Introduction:**

Monogenic diabetes accounts for approximately 1–2% of all diabetes, and is difficult to distinguish from type 1 and type 2 diabetes. Molecular diagnosis is important, as the molecular subtype directs appropriate treatment. Patients are selected for testing according to clinical criteria, but up to 80% of monogenic diabetes in the UK has not been correctly diagnosed. We investigated outcomes of genetic testing in our center to compare methods of selecting patients, and consider avenues to increase diagnostic efficiency.

**Materials and Methods:**

We reviewed 36 probands tested for monogenic diabetes in the last 10 years in a large adult diabetes outpatient clinic, serving an ethnically diverse urban population. We compared published clinical criteria and an online maturity onset diabetes of the young calculator applied to these 36 patients, and presented the predictions together with the molecular results.

**Results:**

The overall mutation detection rate was 42%, reflecting the strict clinical selection process applied before genetic testing. Both methods had high sensitivity for identifying patients with mutations: 88 and 89% for the clinical criteria and online calculator, respectively. Cascade testing in a total of 16 relatives led to diagnosis of a further 13 cases.

**Conclusions:**

Existing patient selection criteria were effective in identifying patients with monogenic forms of diabetes, but the number of patients missed using these strict criteria is unknown. Because of the potential savings resulting from correct molecular diagnosis, it is possible that testing a larger pool of patients using less stringent selection criteria would be cost‐effective. Further evidence is required to inform this assessment.

## Introduction

It is known that 1–2% of patients with diabetes have a monogenic cause of their disease, which can result from mutations in a number of different genes. *HNF1A* and *HNF4A* mutations cause autosomal dominant diabetes, which usually occurs in childhood or early adulthood, and is responsive to treatment with sulphonylureas^1^. Mutations in the *GCK* gene cause autosomal dominant lifelong stable fasting hyperglycemia, which does not require treatment, as patients do not appear to develop the complications of diabetes^2^. *HNF1B* mutations cause diabetes with malformations of the kidney and uterus (renal cysts and diabetes or RCAD syndrome), another dominant condition, which generally requires treatment with insulin^3^. The m.3423A>G mutation in the mitochondrial genome includes diabetes as one of its many phenotypic consequences; this diabetes also requires insulin treatment^4^.

Despite the treatment implications and potential cost savings from making these molecular diagnoses, it was estimated in 2010 that 80% of the approximately 26,000 cases in the UK are currently undiagnosed^5^. Clinical criteria for selecting patients for molecular testing have been produced, but these are detailed and complex because of the heterogeneous nature of monogenic forms of diabetes, and the mutation detection rate when these are applied is unknown^6^. At present, testing rates vary across the UK, indicating a lack of standardization of testing across clinicians and centers^5^.

An online tool is available at http://www.diabetesgenes.org/content/mody-probability-calculator, which has been designed to predict the likelihood of identifying a mutation in the *HNF1A*,* HNF4A* or *GCK* genes using basic clinical information (age at diagnosis, sex, treatment, body mass index, glycated hemoglobin, age and presence or absence of a parent with diabetes), with 75.5% the highest chance possible using this algorithm^7^. Further development of the calculator is ongoing, particularly to include patients from different ethnic backgrounds, as the data used to train the calculator was from individuals of white European origin, as this represents the largest dataset available.

To investigate the effectiveness of published tools for selection of patients for genetic testing, and the outcomes of this testing in our large multi‐ethnic urban diabetes center, we reviewed all genetic tests carried out in adults over the past decade, and present here the results of these tests.

## Materials and Methods

We ascertained the details of patients referred to the Exeter Molecular Genetics Laboratory for monogenic diabetes testing in the 10‐year period from 2004 to February 2014. All samples from the Trust are sent to this laboratory for molecular testing, so this is a complete list of testing arranged by clinicians within the Trust. All of the patients included in the study attended the diabetes outpatient clinic at Guy's and St Thomas’ NHS Foundation Trust on at least one occasion between 2004 and 2014, having been referred by their general practitioner. Genetic testing was requested when the clinician's index of suspicion for a monogenic form of diabetes was high.

The pathogenicity of the mutations identified during genetic testing was assessed by the Exeter Molecular Genetics Laboratory, which is one of the principle centers for molecular diagnosis of diabetes worldwide. In‐house expertise was combined with application of the Practice Guidelines for the Evaluation of Pathogenicity and the Reporting of Sequence Variants in Clinical Molecular Genetics, produced by the United Kingdom Association for Clinical Genetic Science (which can be viewed at http://www.acgs.uk.com/committees/quality-committee/best-practice-guidelines).

Proband diagnostic testing and cascade testing in relatives were analyzed separately. Paper and electronic records were reviewed to obtain clinical information for probands, including date of diagnosis, treatment, body mass index, family history, biochemical parameters (HbA1c, antibody testing, C‐peptide) and ethnic origin.

The principal criteria given in the 2008 Consensus Criteria for identification of *HNF1A* and *HNF4A* monogenic diabetes were onset of diabetes below age 25 years in one family member, presence of diabetes in two consecutive generations, absence of pancreatic auto‐antibodies within 3 years of onset of diabetes and evidence of continuing endogenous insulin after 3 years of diabetes^6^. These criteria were assessed in the probands who had undergone genetic testing. Antibodies tested included either or both of GAD and ICA. Evidence of ongoing insulin production included patients not receiving insulin treatment, or patients with C‐peptide greater than 200 pmol/L after 3 years on insulin.

Clinical data were also entered into the online maturity onset diabetes of the young (MODY) calculator for patients diagnosed with diabetes below the age of 35 years to generate a percentage prediction of the chance of detecting a mutation in *HNF1A*,* HNF4A* or *GCK*; the tool has not been validated for patients diagnosed after this age.

## Results

A total of 79 probands in the study center underwent genetic testing for genes causing monogenic diabetes between 2004 and February 2014. Of these, 36 probands were seen in the adult diabetes clinic, and were tested for one or more of the monogenic diabetes genes *GCK*,* HNF1A*,* HNF4A* and *HNF1B* or the mitochondrial m.3243A>G mutation. A total of 28 of these tests (78%) were requested in the last 5 years since 2009. A total of 15 patients had a molecular diagnosis identified (42%): five *HNF1A*, five m.3243A>G, three *HNF4A* and two *GCK*. Clinical characteristics of the probands are given in Table 1. The remaining 43 probands were tested in a pediatric clinic, a genetics clinic or a renal clinic; one patient had an *HNF1A* mutation, another a *KCNJ11* mutation and nine had mutations in *HNF1B*. The focus of this study was genetic testing patterns and outcomes within the adult diabetes center, and the remainder of this report therefore focuses on the 36 probands tested in the adult diabetes clinic.

**Table 1:** Clinical characteristics of probands who underwent genetic testing

	Sex		Ethnicity			Median age at testing, years (range)	Median years from diagnosis to test (range)	Average age of onset (years)	Average BMI	Delayed/no insulin requirement (%)
	M	F	White European	Afro‐Caribbean	Other					
*HNF1A*/*HNF4A*	2	6	6	0	2	34 (18–51)	17 (0–31)	20.0	26.6	88
*GCK*	2	0	2	0	0	35 (32–37)	0	34.5	25.7	100
m.3243A>G	1	4	2	3	0	44 (31–53)	21 (0–25)	27.2	22.8	20
No mutation	13	8	14	1	6	36 (15–68)	12 (0–53)	23.7	27.7	62
All patients	18	18	24	4	8	35 (15–68)	15 (0–53)	24.0	26.7	72

Patient numbers are too small to draw formal statistical conclusions regarding the association between these clinical characteristics and the presence or absence of the different types of mutation. However, it is notable that the patients with mitochondrial diabetes tended to require insulin earlier than those with *HNF1A* and *HNF4A* diabetes, which is consistent with results from larger cohorts of patients^4^. All 36 patients had at least one close family member with diabetes.

Of the adult diabetes patients with no molecular diagnosis, seven had complete testing of *HNF1A* and *HNF4A* including multiplex ligation‐dependent probe amplification (MLPA) for deletions and duplications. Nine probands had partial testing of these genes; these patients were no longer attending the clinic and it was therefore not possible to complete this testing. Four of the patients with no molecular diagnosis were only tested for the mitochondrial mutation, as their phenotype was not thought to be compatible with *HNF1A* or *HNF4A* diabetes. Most patients were not tested for *GCK*, as this was only requested in patients with mildly raised fasting glucose.

**Figure 1:**
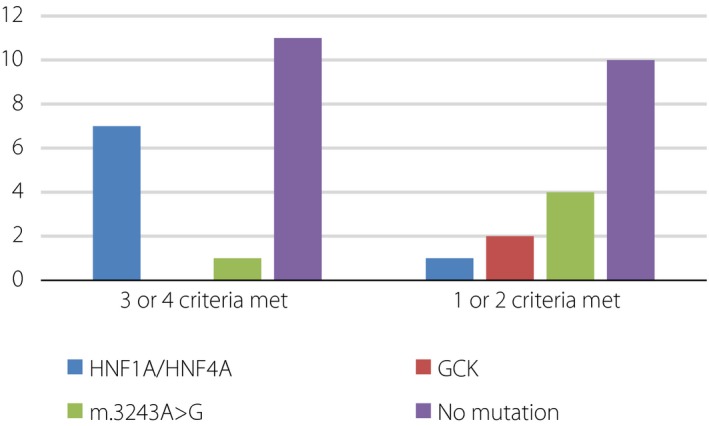
Mutation detection according to the 2008 consensus criteria: in patients meeting three or four or the criteria, seven of 19 (37%) had a mutation in *HNF1A* or *HNF4A*, whereas one (5%) had a mitochondrial mutation. In patients meeting only one or two of the criteria, one of 17 (5%) had a mutation in *HNF1A* or *HNF4A*, whereas six of 17 (35%) had a mutation in other genes.

Probands were assessed according to the 2008 Consensus criteria for identification of patients with *HNF1A* and *HNF4A* mutations^6^. Evidence was not available to allow assessment of all four categories in all probands. Three probands met all four criteria; one of these had an *HNF4A* mutation. A total of 16 probands met three out of four criteria; four of these had an *HNF1A* mutation, two had an *HNF4A* mutation and one had the mitochondrial mutation. The remaining 18 probands met one or two of the criteria based on available information; these included one patient with an *HNF1A* mutation, two with *GCK* mutations and four with the mitochondrial mutation (Figure 1).

Only one tested patient had positive autoantibodies, and this patient had no mutation detected. Mutations were found in individuals with negative antibodies at the time of diagnosis (three mutations in 11 patients tested), in patients with negative antibodies more than 3 years after diagnosis (eight of 13 patients) and in those without antibody results (four of 11 patients).

Positive C‐peptide is used to assess ongoing endogenous insulin secretion, and is likely to be present in patients with diabetes caused by *HNF1A* and *HNF4A* mutations. Mutations in these genes were found in six of 21 patients with positive C‐peptide, no patients with low C‐peptide, and two of 13 patients where C‐peptide was not measured.

Overall, of the eight patients with mutations in *HNF1A* and *HNF4A*, seven met three of four of the 2008 consensus criteria. For the final patient, insufficient information was available regarding ages of onset within the family and biochemical testing to assess the patient against these criteria. A total of 12 patients who met three or four of the criteria did not have a mutation in *HNF1A* or *HNF4A*, giving a mutation detection rate of seven out of 19 (37%) using this tool, with sensitivity 88% and specificity 57% (Table 2).

**Table 2:** Performance of the 2008 Consensus criteria and online MODY calculator in predicting patients with a mutation in *HNF1A* or *HNF4A* (and *GCK* for the MODY calculator)

2008 criteria	3/4 criteria met	0–2 criteria met	
Mutation	7	1	Sensitivity 88%
No mutation	12	16	Specificity 57%
	PPV 37%	NPV 94%	

MODY calculator	Predicted >20%	Predicted <20%	
Mutation	8	1	Sensitivity 89%
No mutation	8	14	Specificity 64%
	PPV 50%	NPV 93%	

MODY, maturity onset diabetes of the young; NPV, negative predictive value; PPV, positive predictive value.

**Figure 2:**
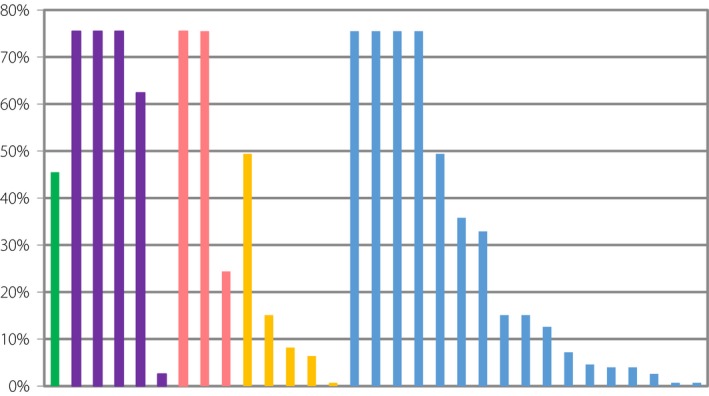
Results from the maturity onset diabetes of the young probability calculator divided by molecular diagnosis: green bar, *GCK*; purple bar, *HNF1A*; pink bar, *HNF4A*; yellow bar, m.3243A>G; blue bar, no mutation identified. The calculator has been trained to detect individuals with *GCK*,*HNF1A* and *HNF4A* mutations; patients with mitochondrial diabetes are shown here for purposes of comparison along with the patients in whom no mutation was identified.

All probands were also assessed using the online MODY calculator; the predictions are shown divided by molecular result in Figure 2. Overall, 31 of 36 patients could be assessed using the tool, as five probands were over the age of 35 years at diagnosis, which falls outside the range that can be calculated using this tool. A total of 16 patients were predicted to have a greater than 20% chance of detecting a mutation; of these, eight had a mutation detected in *HNF1A*,* HNF4A* or *GCK* (50%). A total of 15 patients had prediction scores less than 20%, of which one had a mutation in one of the three genes, giving a sensitivity of 89% and specificity 64% using the 20% threshold (Table 2).

One patient with diabetes diagnosed in early childhood had a close family history of the m.3243A>G mutation, and therefore this patient's diabetes was assumed to be mitochondrial in origin, despite the test in blood failing to show the mutation. This illustrates the importance of testing multiple tissues, and considering family history and phenotype when diagnosing mitochondrial diabetes, as the mutation might not be present in the blood, particularly in adults.

Cascade testing was carried out in 16 individuals from five families (Table 3). All 11 relatives with diabetes who underwent testing carried the familial mutation, as expected. Of the five relatives without diabetes who underwent predictive testing, three did not carry the familial mutation, releasing these individuals and their offspring from the necessity for ongoing surveillance for diabetes.

**Table 3:** Cascade tests carried out in 16 members of five families

Family	Gene	Relatives with diabetes	Relatives without diabetes
1	m.3243A>G	1 (1 pos)	0
2	*HNF1A*	3 (3 pos)	1 (1 pos)
3	*HNF4A*	4 (4 pos)	2 (1 pos)
4	*HNF4A*	1 (1 pos)	0
5	*HNF4A*	2 (2 pos)	2 (0 pos)
Total		11 (11 pos)	5 (2 pos)

The total number of tests is shown for relatives with diabetes (diagnostic cascade testing) and relatives without diabetes (predictive cascade testing), including the number of positive tests in brackets (pos).

Of the eight probands and five relatives with diabetes seen in our center diagnosed with *HNF1A* or *HNF4A* diabetes, three were prescribed a sulphonylurea drug at the time of molecular testing. At the most recent review, ten patients were prescribed a sulphonylurea; of the remaining three patients, one declined a trial of oral medication, and two were treated with diet only. Eight of the ten patients on a sulphonylurea required insulin treatment in addition to oral hypoglycaemic agents. Four patients were pregnant or attempting to conceive at the time of diagnosis or follow up, and their management was therefore altered accordingly.

## Discussion

Monogenic diabetes is an underdiagnosed condition with significant implications for patient management. We have presented our experience of selecting patients for genetic testing over the past decade. A total of 28 patients from 15 families received a molecular diagnosis during this time. These diagnoses allowed patients to understand their chance of passing on their condition to their children, assisted with cascade testing in other family members and affected clinical management of diabetes. This was particularly relevant in female patients of childbearing age, as an awareness of the diagnosis allowed specific management of maternal diabetes as well as preparation for the possibility of neonatal complications.

Nine patients who were suspected to have *HNF1A* or *HNF4A* mutations did not receive full sequencing and dosage analysis by MLPA for both genes. Before April 2013, the diagnostic laboratory used a staged testing process with different levels of testing attracting different costs. The levels include testing a single common mutation in *HNF1A*, sequencing of one or both genes, and MLPA testing for deletions and duplications within both genes. The report format included this information, but in some cases the headline of the report stating that no mutation had been found appears to have been interpreted to mean that complete testing had been carried out, when not all levels of testing had been completed, despite a high predicted chance of finding a mutation. This illustrates the complexity of arranging and interpreting genetic testing, where the residual chance of a false negative result might be difficult to appreciate. The testing process is now much simpler, so the results issued since April 2013 are easier to interpret.

Mutation detection rates have been reported to vary between different populations, with higher pick‐up rates in European studies, and lower rates in South and East Asian studies^8^, ^9^. Very few studies of Afro‐Caribbean populations have been published. Our center caters for a population of high ethnic variability, which is reflected in the patients reported here. In this cohort, mutations were identified in patients from a wide variety of ethnic backgrounds; larger numbers would be required to detect an effect of ethnicity on mutation rate.

Cascade testing only occurred within the UK in five of the 15 families in whom a proband received a molecular diagnosis. In part, this reflects the nature of the urban population, with many patients having no relatives within our area, or even in the UK. However, it must be noted that the resources of time and expertise required for cascade testing in terms of contacting families, and arranging appropriate referrals and testing are not readily available in busy mainstream diabetes outpatient clinics. This situation is addressed in our center by the dedicated Monogenic Diabetes clinic, with input from specialists in diabetes and clinical genetics, and specialist diabetes nurses.

Diabetes caused by *HNF1A* and *HNF4A* mutations is classically responsive to sulphonylurea treatment. In this patient cohort, patients were appropriately offered sulphonylurea treatment after molecular diagnosis, but most patients still required insulin in addition to oral hypoglycemic agents. This is likely to reflect several factors: first, four of the 13 patients were pregnant or attempting to conceive, which affected the management options available; second, molecular diagnoses in this cohort in many cases followed many years after onset of diabetes, and in the longer term many *HNF1A* and *HNF4A* patients do progress to requiring insulin; and third, intensive education and support is required to transfer patients off insulin, and this is complicated by higher rates of patient mobility, language barriers and low socioeconomic status in urban diabetes centers. Collection of real‐world data regarding treatment alteration after molecular diagnosis will be required to inform health economic assessment of the cost‐effectiveness of genetic testing in diabetes.

The characteristics of tested probands were assessed using the 2008 consensus criteria (which are directed at identifying *HNF1A* and *HNF4A* mutation patients) and the online MODY calculator (trained to identify patients with *HNF1A*,* HNF4A* and *GCK* mutations)^6^, ^7^. Patients with mitochondrial and *HNF1B* diabetes can be more readily detected through their clinical and biochemical features. A comparison of the performance of the two tools is given in Table 2, showing that both tools give good discrimination between patients with and without mutations. However, it is not known what proportion of individuals with diabetes would need to be tested if these criteria were widely applied, and therefore whether the cost associated with these thresholds would be manageable within NHS budgets.

Using the online MODY calculator, eight of nine patients with *HNF1A, HNF4A* or *GCK* mutations scored over 20%. The patient with an *HNF1A* mutation with a score of 2.6% on the calculator would have scored 75.5% if she had not been treated with insulin within 6 months of diagnosis. This patient was diagnosed in her teens in the early 1990s, when all slim adolescent patients with diabetes would have been assumed to have type 1 diabetes and automatically treated with insulin. This illustrates the need for caution when using the MODY calculator for patients who were diagnosed before awareness of monogenic diabetes was widespread, as the calculator is very sensitive to the timing of insulin treatment. Of the four individuals with prediction scores of 75.5% in whom no mutations were identified, two had complete testing of *HNF1A* and *HNF4A* including MLPA, whereas two did not.

Our experience (albeit with small patient numbers) suggests that a high score using available tools does yield a high mutation detection rate (42% overall in this cohort). However, experience in other monogenic subsets of complex diseases (e.g., familial hypercholesterolemia, breast cancer) has shown that the higher the specificity of the test the lower the sensitivity. This means that using these strict criteria is likely to miss a proportion of affected patients. A limitation of the present study was that standardized criteria were not in use in this clinic during this period to identify patients suitable for genetic testing. This means that the total number of patients seen in the clinic who would have met these testing criteria is unknown. This is being addressed prospectively by the implementation of standardized testing criteria.

At present, there are no health economic assessments based on real‐world evidence to determine how stringent the criteria should be to maximize the cost‐effectiveness of the testing, but given the potential cost savings of making these diagnoses and tailoring treatment for all family members, it is possible that testing more widely would be cost‐effective overall. Applying less stringent criteria, testing larger numbers of patients and reducing the mutation detection rate is the only method to discover how many patients are missed by applying these strict criteria. With the costs of genetic testing falling, and the continuing drive to increase awareness of monogenic diabetes, this increase in testing is a realistic goal, which needs to be supported by the development of evidence‐based guidelines and health economic assessments to standardize testing, and focus test resources equitably and effectively.

## Disclosure

The authors declare no conflict of interest.
